# Symptoms, signs and nerve conduction velocities in patients with suspected carpal tunnel syndrome

**DOI:** 10.1186/1471-2474-14-242

**Published:** 2013-08-15

**Authors:** Georgia Ntani, Keith T Palmer, Cathy Linaker, E Clare Harris, Richard Van der Star, Cyrus Cooper, David Coggon

**Affiliations:** 1 MRC Lifecourse Epidemiology Unit, University of Southampton, Southampton SO16 6YD, UK; 2 Department of Clinical Neurophysiology, Wessex Neurological Centre, Southampton General Hospital, Southampton, UK

**Keywords:** Epidemiology, Evidence-based medicine, Hand, Nerve compression syndromes, Wrist

## Abstract

**Background:**

To inform the clinical management of patients with suspected carpal tunnel syndrome (CTS) and case definition for CTS in epidemiological research, we explored the relation of symptoms and signs to sensory nerve conduction (SNC) measurements.

**Methods:**

Patients aged 20–64 years who were referred to a neurophysiology service for investigation of suspected CTS, completed a symptom questionnaire (including hand diagrams) and physical examination (including Tinel’s and Phalen’s tests). Differences in SNC velocity between the little and index finger were compared according to the anatomical distribution of symptoms in the hand and findings on physical examination.

**Results:**

Analysis was based on 1806 hands in 908 patients (response rate 73%). In hands with numbness or tingling but negative on both Tinel’s and Phalen’s tests, the mean difference in SNC velocities was no higher than in hands with no numbness or tingling. The largest differences in SNC velocities occurred in hands with extensive numbness or tingling in the median nerve sensory distribution and both Tinel’s and Phalen’s tests positive (mean 13.8, 95% confidence interval (CI) 12.6-15.0 m/s). Hand pain and thumb weakness were unrelated to SNC velocity.

**Conclusions:**

Our findings suggest that in the absence of other objective evidence of median nerve dysfunction, there is little value in referring patients of working age with suspected CTS for nerve conduction studies if they are negative on both Tinel’s and Phalen’s tests. Alternative case definitions for CTS in epidemiological research are proposed according to the extent of diagnostic information available and the relative importance of sensitivity and specificity.

## Background

Carpal tunnel syndrome (CTS) arises from entrapment of the median nerve at the wrist, which if necessary can be treated by surgical decompression. Typically the disorder is characterised by numbness or tingling in the sensory distribution of the nerve in the hand, which in some cases may be accompanied by pain, or by weakness of the muscles of thumb abduction and opposition. Pointers to the diagnosis include positive findings on challenge tests in which either tapping over the median nerve at the wrist (Tinel’s test) or sustained flexion of the wrist (Phalen’s test) produce numbness, tingling or pain in the nerve’s sensory distribution. However, neurophysiological demonstration of impaired conduction in the median nerve is widely regarded as a more reliable indicator of the disease [1].

That said, nerve conduction studies cannot be regarded as a gold standard for diagnosis since the correlation of symptoms with abnormal nerve conduction, however defined, is imperfect [2-7]. Thus, there are no universally agreed diagnostic criteria for CTS, and decisions to carry out surgical decompression may depend on a combination of symptoms, signs and findings from nerve conduction studies, according to the practice of the surgeon [1,8]. Nevertheless, in managing patients with symptoms suggestive of CTS, it would be useful to know which clinical features are most predictive of impaired nerve conduction, and whether there are combinations of clinical findings for which nerve conduction is no different from that in asymptomatic hands.

Away from clinical practice, there is also a need to optimise diagnostic criteria for CTS in epidemiological research. In these circumstances, a different approach may be required. For example, in population surveys, nerve conduction studies may not be possible, or may be practical for only a subset of participants. How should cases be defined when information is available only about symptoms, or only about symptoms and physical signs, and on what basis should subjects be selected for nerve conduction studies? If nerve conduction studies are available, what should be the criteria for abnormality? Again, answers to these questions will depend on the relation of symptoms and signs to nerve conduction measurements.

A number of previous studies have examined the relationship of clinical findings to abnormalities of nerve conduction [6,9-30], but most have been small [10-12,15-18,20-22,24-28] or have assessed associations with pre-defined composite scores without attempting to disentangle the relative importance of different component symptoms and signs [10,20-23,29]. Moreover, with few exceptions [11,24,27,28,30,31] they have classified nerve conduction dichotomously and have not compared distributions of measurements with the distribution found in asymptomatic hands.

To obtain more detailed information on the relation of clinical findings to nerve conduction, we analysed data from a consecutive series of patients referred to a clinic for neurophysiological investigation because of suspected CTS. Our two aims were to establish whether there are combinations of symptoms and signs for which nerve conduction studies are very unlikely to be abnormal and therefore are of dubious value, and to inform choices of case definition for use in epidemiological studies. In contrast to most previous studies, we analysed detailed symptoms and signs individually rather than as composite scores, and made no a priori assumptions about the level of impairment of median nerve conduction that should be classed as abnormal.

## Methods

As part of a larger study, all patients aged 20–64 years, who attended the neurophysiology service at Southampton General Hospital during January 2007 to September 2009 for investigation of suspected CTS, were sent a letter inviting them to complete a self-administered questionnaire in advance of their hospital appointment.

Among other things, the questionnaire asked about sex, age, and the occurrence of numbness, tingling and pain in each hand during the past four weeks. Those who had experienced symptoms were asked to mark their anatomical distribution on hand diagrams (one diagram for each symptom) [20], and also the number of days during the past four weeks, if any, on which the symptoms had disturbed their sleep.

When patients attended hospital they were seen by a research nurse (CL), who collected completed questionnaires (in a few cases where the patient had failed to complete part or all of the questionnaire, he or she was asked to do so in the clinic or to return it later), and carried out a brief physical examination of the hands. This comprised an assessment of the strength of abduction and opposition of the thumb and performance of Tinel’s and Phalen’s tests. At the time of the physical examination, neither the patient nor the nurse knew whether the neurophysiological examination was considered abnormal.

In the assessment of muscle strength, the patient was asked to place the dorsum of each hand in turn, flat on a surface, and to maintain the thumb in abduction against a force which the nurse applied with her thumb. The patient was then asked to oppose the tips of the thumb and little finger while the nurse applied a counter-force with her thumb and index finger. Weakness was considered to be present if the patient could not maintain a posture when challenged.

Tinel’s test was performed by percussing the wrist briskly three times with a tendon hammer over the flexor retinaculum, just radial to the palmaris longus tendon at the distal wrist crease. In Phalen’s test, the patient was asked to maintain the wrists in 90° flexion for one minute, with the backs of the fingers opposed. In each case, the test was deemed positive if it provoked numbness, tingling or pain in the thumb, index finger, middle finger or medial palmar surface of the hand.

Each nerve conduction study was carried out using a Nicolet machine by a physician or clinical physiologist trained in clinical neurophysiology. Sensory and motor nerve conduction tests were performed to look for evidence of carpal tunnel syndrome in accordance with the normal departmental protocol. Among other things, measurements were made of orthodromic sensory nerve conduction (SNC) from index, middle and little fingers to wrist, with surface recordings made over the median or ulnar nerves proximal to the distal wrist crease. With the patient’s permission, the findings were subsequently abstracted by the research nurse from the clinical record.

Statistical analysis was carried out using Stata version 11.1 [32], and was restricted to hands which had not previously been treated surgically for CTS. As a first step, we wished to identify a single continuous measure of median nerve function to which clinical findings could be related. It is widely accepted that in CTS, abnormality of sensory nerve conduction is seen before abnormality of motor nerve conduction, and we therefore focused on the former. To determine a suitable measure of sensory nerve conduction, we explored the frequency distributions and inter-correlations of three alternative indices – SNC velocity in the index finger, SNC velocity in the middle finger, and the difference between SNC velocity in the little and index fingers.

**Figure 1:**
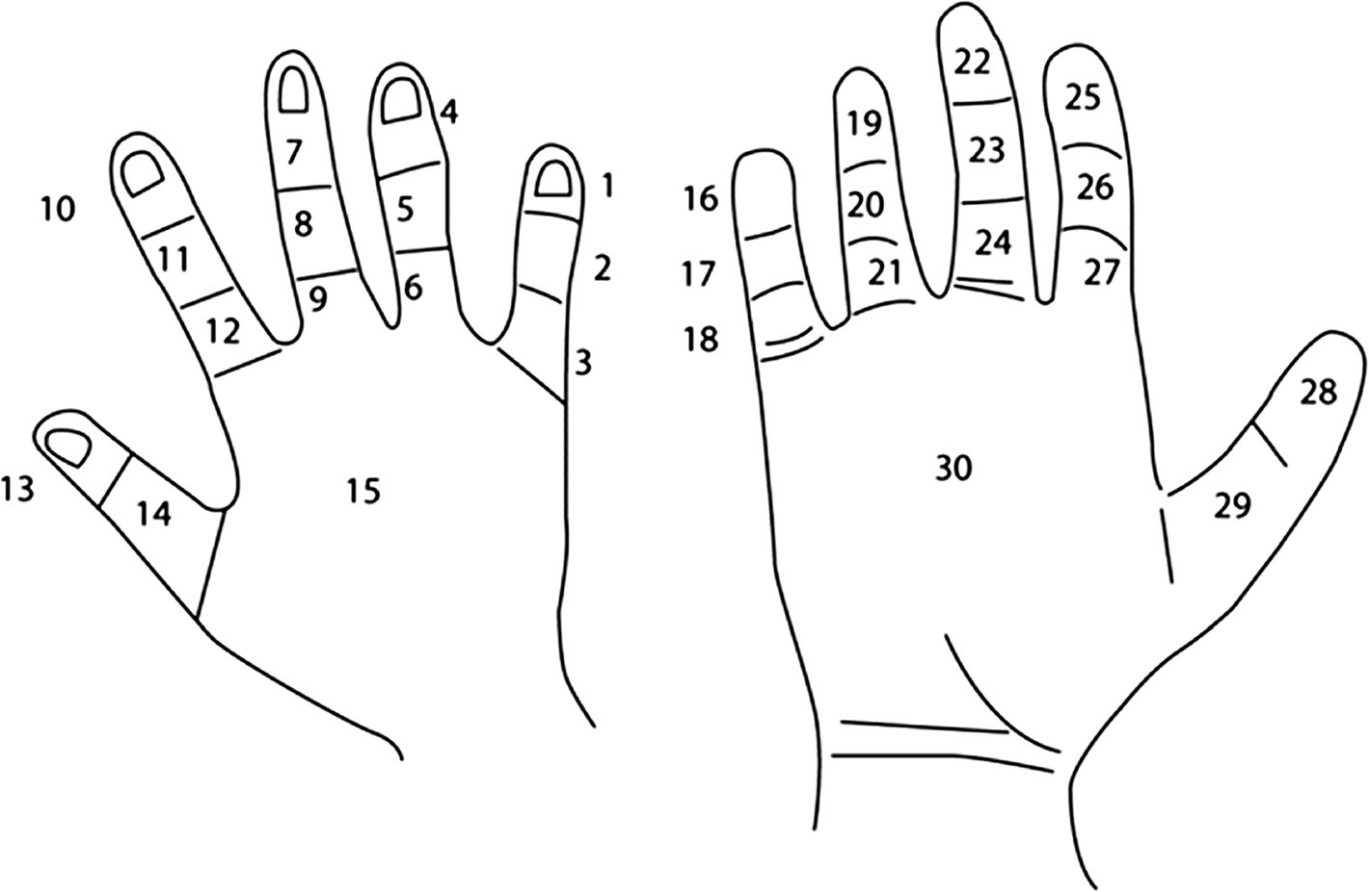
Classification of anatomical regions of hand.

Having chosen a suitable measure of nerve conduction, we next examined its relationship to symptoms and findings on physical examination. For this purpose, the completed hand diagrams were used to characterise the distribution of numbness/tingling and pain in each hand, according to which of 30 anatomical regions were affected (Figure 1). A region was considered positive for numbness/tingling if it was affected by either symptom. Each of the 30 regions was classified according to whether it fell exclusively within the sensory distribution of the median nerve (regions 22–29), partly within its distribution (regions 7, 10, 19–21 and 30), or entirely outside it (regions 1–6, 8–9 and 11–18). These categories were designated “median”, “part-median” and “non-median”. As in an earlier study [33], symptoms that occurred in the median area were sub-classified as “extensive median” or “limited median”, according to whether the number of regions 22–29 with symptoms was ≥6 or <6. Means and standard deviations of SNC velocity were derived for different symptom patterns and examination findings individually, and then their mutually adjusted associations with SNC velocity were assessed by multiple linear regression. This analysis adjusted also for sex and age, and used random effects modelling to allow for the possibility that results for two hands from the same patient were not independent. We then derived mean SNC velocities for combinations of symptoms and clinical findings which the regression analysis suggested were most predictive of abnormality.

To identify a suitable cut-point in the distribution of SNC velocities by which cases might be defined in epidemiological research, we compared the distribution of SNC velocities in hands with clinical findings which the above analysis indicated were associated with the lowest SNC velocities, and that in hands with no symptoms or physical signs. This analysis was carried out in a random 50% subset of hands, and then the performance of the proposed cut-point in relation to clinical findings was assessed in the other 50% of hands.

Ethical approval for the study was provided by Southampton and South-West Hampshire NHS Research Ethics Committee.

## Results

**Figure 2:**
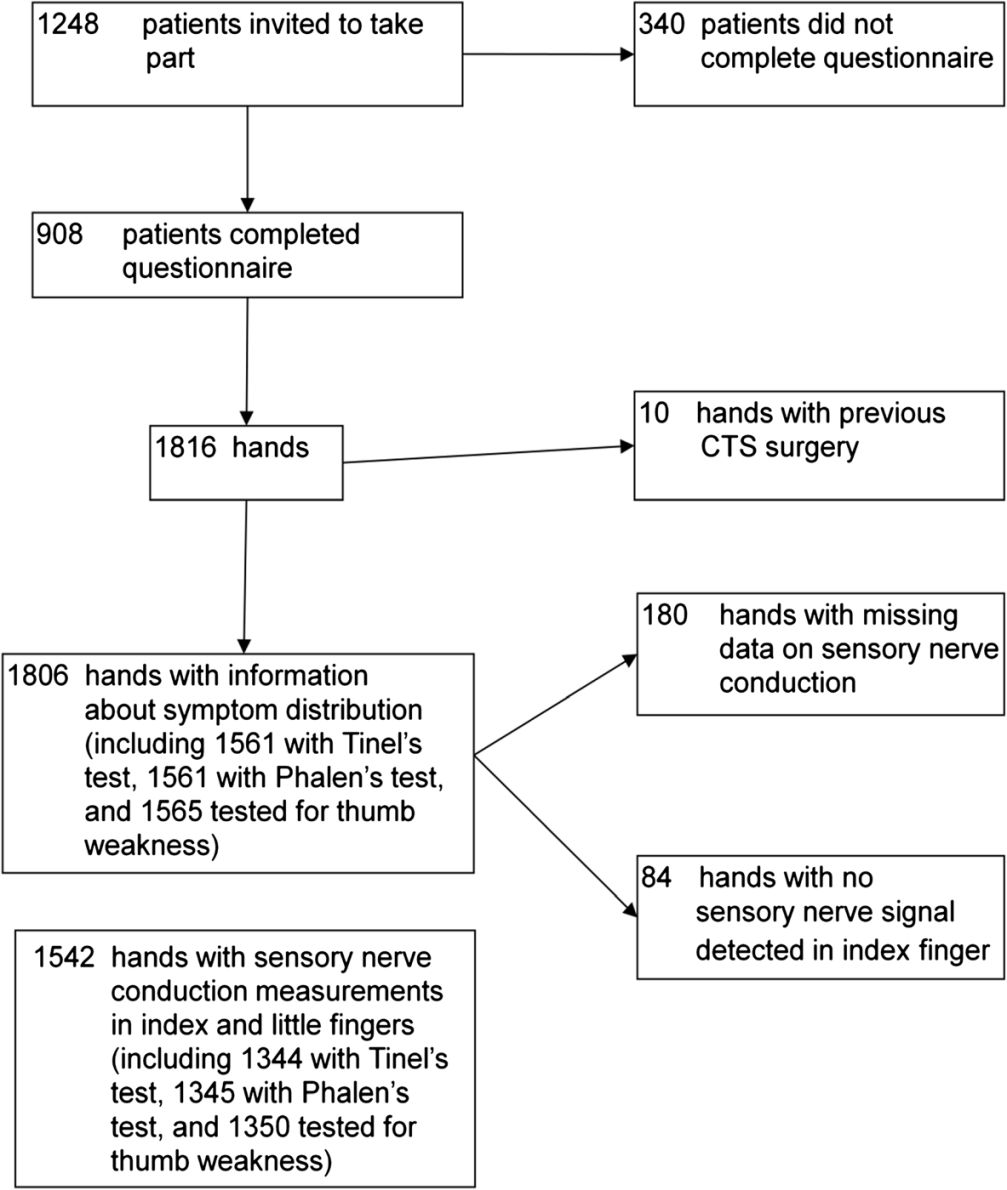
Recruitment of patients and numbers of hands analysed.

A total of 1248 patients were invited to take part in the study, of whom 908 (73%) completed questionnaires – 298 men and 610 women with mean age 47.1 years, median 48.0 years and inter-quartile range 39.8 to 55.1 years. These patients provided information on 1816 hands, but 10 hands were excluded from analysis because of previous surgery for CTS (Figure 2).

Among the remaining 1806 hands, 1571 (87%) provided satisfactory measurements of SNC for the index finger, 1302 (72%) for the middle finger, and 1627 (90%) for the little finger. For each digit, the distribution of SNC velocities was approximately normal. The three measures of SNC velocity investigated were all highly correlated, with a particularly close relation between SNC velocity in the index and middle fingers (r = 0.97). In subsequent analyses, the difference in SNC velocities between the little and index fingers was adopted as the preferred continuous measure of median nerve function since it provided control for possible residual effects of variation in hand temperature, and because data were more complete for the index than the middle finger.

Table 1 summarises the distribution of numbness/tingling and pain in the 1806 hands that were analysed. Most hands (1459) had been affected by numbness or tingling in the month before completing the questionnaire, but fewer (893) had been painful. Most often, numbness/tingling was reported in all three of the median, part-median and non-median regions (including 787 hands with extensive median involvement and 286 with limited median involvement). In contrast, limitation of numbness and tingling to the median and/or part-median regions with no involvement of the non-median regions was much less common (216 hands). On the basis of this analysis, the observed patterns of symptoms were assigned to groups (eight for numbness/tingling and five for pain) in a way that ensured adequate numbers of hands in each group (where a symptom distribution occurred in only a few hands, it was aggregated with another similar distribution). The definition of these groups is indicated in Table 1.

**Table 1:** Distribution of sensory symptoms in hand and definition of symptom groups

**Affected regions of hand**			**Numbness or tingling**		**Pain**	
**Median**	**Part-median**	**Non-median**	**Number of hands**	**Group**	**Number of hands**	**Group**
No	No	No	347	0	913	0
No	No	Yes	31	1	91	1
No	Yes	No	11	2	53	2
No	Yes	Yes	126	2	127	2
Limited	No	No	32	3	12	3
Limited	No	Yes	11	4	26	3
Limited	Yes	No	63	5	65	3
Limited	Yes	Yes	286	4	215	3
Extensive	No	No	14	7	1	4
Extensive	No	Yes	2	6	1	4
Extensive	Yes	No	96	7	23	4
Extensive	Yes	Yes	787	6	279	4

Table 2 shows differences in SNC velocities between the little and index fingers (mean and standard deviation) for different distributions of symptoms, and according to findings on physical examination of the hand. Data on physical examination (Tinel’s and Phalen’s tests, thumb weakness) were missing for 235 hands because the patient attended hospital on a day when the research nurse was unavailable, and were incomplete for a few further hands. Differences in SNC velocity were higher in hands classed to Groups 6 and 7 for numbness/tingling (i.e. those with extensive median involvement). Positive Tinel’s and Phalen’s tests were also associated with impaired median nerve conduction, but there was no clear relation of median nerve conduction to pain or to thumb weakness. When associations with clinical findings were mutually adjusted in a multiple linear regression analysis, the associations with Groups 6 and 7 for numbness/tingling, and with positive Tinel’s or Phalen’s tests were all statistically significant (Table 2).

**Table 2:** Relation of clinical findings to difference in sensory nerve conduction velocity between little and index finger

**Clinical finding**	**Number of hands**	**Number of hands with nerve conduction measurements**	**Mean (SD) difference in nerve conduction velocity (m/s)**	**Linear regression analysis**	
				**Regression coefficient**^**a**^	**95% CI**
**Numbness/ tingling group**					
0	347	221	6.4 (7.4)	Baseline	-
1	31	31	4.0 (5.9)	−0.9	−3.8 to 2.1
2	137	128	6.4 (9.7)	0.2	−1.5 to 2.0
3	32	27	3.4 (6.0)	−1.7	−4.7 to 1.3
4	297	272	7.6 (9.6)	0.8	−0.6 to 2.2
5	63	55	6.4 (8.2)	1.3	−0.9 to 3.5
6	789	708	10.1 (9.4)	2.8	1.5 to 4.0
7	110	100	12.0 (10.3)	3.7	1.8 to 5.6
**Pain group**					
0	913	731	8.8 (9.3)	Baseline	-
1	91	83	5.8 (9.1)	−0.3	−2.2 to 1.6
2	180	172	8.0 (9.0)	0.2	−1.2 to 1.5
3	318	291	8.4 (9.4)	−0.7	−1.9 to 0.5
4	304	265	9.4 (9.5)	0.2	−1.1 to 1.5
**Tinel’s test**					
Negative	1110	949	7.2 (8.7)	Baseline	-
Positive	451	395	12.4 (9.7)	2.5	1.6 to 3.5
Missing	245	198	7.9 (9.5)	−1.6	−6.2 to 2.9
**Phalen’s test**					
Negative	696	574	5.2 (7.7)	Baseline	-
Positive	865	771	11.2 (9.6)	3.3	2.3 to 4.3
Missing	245	197	8.0 (9.5)	3.9	−2.2 to 9.9
**Thumb weakness**^**b**^					
Negative	1403	1218	8.6 (9.2)	Baseline	-
Positive	162	132	9.2 (10.0)	−0.7	−2.1 to 0.7
Missing	241	192	8.1 (9.5)	−0.4	−7.3 to 6.4

^a^Adjusted for sex, age and other variables in table.

^b^Weakness of abduction or opposition.

We next examined nerve conduction velocities for combinations of symptoms and signs which the multiple regression analysis had indicated were most predictive of abnormality. For this purpose, distributions of numbness and tingling were further aggregated as shown in Table 3. In hands with no numbness or tingling and negative on both Tinel’s and Phalen’s test, the mean difference in SNC velocity between the little and index finger was 5.0 m/s. Relative to this value, differences in SNC velocities were materially increased only when Tinel’s or Phalen’s test was positive, the highest difference in velocities being found in hands with extensive median numbness/tingling and both Tinel’s and Phalen’s tests positive (mean difference 13.8, 95% confidence interval (CI) 12.6 to 15.0 m/s).

**Table 3:** Difference between sensory nerve conduction velocities in the little and index fingers according to combinations of clinical findings

**Numbness/tingling group**^**a**^	**Tinel’s test**	**Phalen’s test**	**Number of hands**	**Aggregate category**	**Number of hands with nerve conduction measurements**	**Mean (95% CI) difference between SNC**^**b **^**velocities in the little and index fingers (m/s)**
0	Negative	Negative	232	A	144	5.0 (3.9 to 6.1)
0	Negative	Positive	40	B	51	9.7 (7.7 to 11.8)
0	Positive	Negative	8	B		
0	Positive	Positive	18	B		
1, 2	Negative	Negative	76	C	75	3.6 (1.9 to 5.3)
1, 2	Negative	Positive	40	D	60	8.9 (6.3 to 11.5)
1, 2	Positive	Negative	2	D		
1, 2	Positive	Positive	22	D		
3-5	Negative	Negative	137	E	127	3.3 (2.1 to 4.5)
3-5	Negative	Positive	106	F	106	8.7 (6.9 to 10.5)
3-5	Positive	Negative	9	F		
3-5	Positive	Positive	85	G	72	10.6 (8.4 to 12.9)
6-7	Negative	Negative	195	H	177	6.6 (5.3 to 7.8)
6-7	Negative	Positive	275	I	282	10.6 (9.5 to 11.6)
6-7	Positive	Negative	32	I		
6-7	Positive	Positive	274	J	241	13.8 (12.6 to 15.0)

^a^For definitions of groups see Table 1.

^b^Sensory nerve conduction.

**Figure 3:**
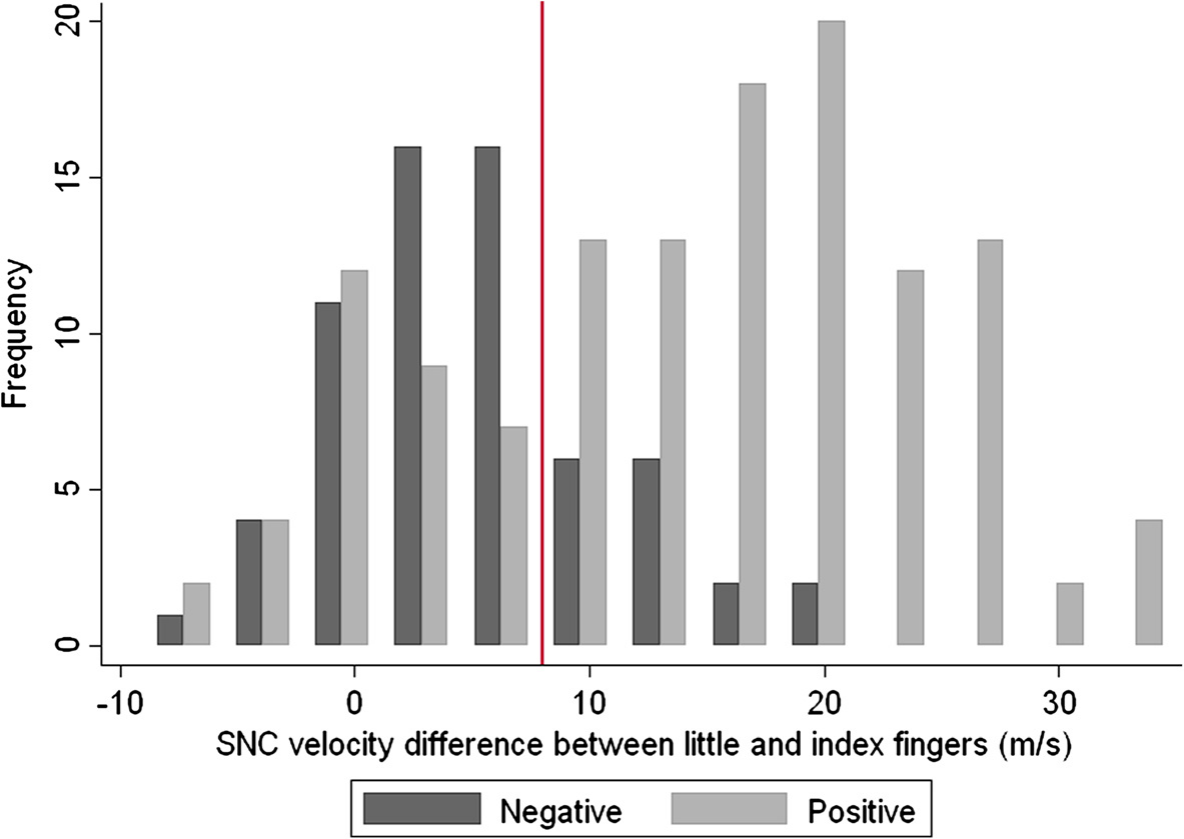
Distributions of differences in sensory nerve conduction velocity between the little and index fingers in a random 50% sample (N=193) of hands a) with no numbness/tingling and negative for Tinel’s and Phalen’s tests ("negative" hands) or b) positive for all three of these clinical features ("positive" hands). The vertical red line indicates the proposed cut-point for abnormality of sensory nerve conduction.

To derive a cut-point for abnormality of the difference in SNC velocities between the little and index fingers that might be used in epidemiological studies, we compared the distribution of measurements in hands which had extensive median numbness/tingling and were positive on both Tinel’s and Phalen’s tests, with that in hands which had no numbness or tingling and were negative on both tests. For this analysis we used a 50% random subset (n=193) of the hands that met these clinical criteria. As illustrated in Figure 3, there was overlap between the two distributions, a number of clinically positive hands having differences in SNC velocities less than 5 m/s. However, the modal values were distinct, and a value of 8 m/s appeared to discriminate between the two sets of hands reasonably well. When this cut-point was applied in the other random 50% of hands, the prevalence of abnormality (i.e. difference in SNC velocity > 8 m/s) was 25% in hands with no numbness/tingling and negative on Tinel’s and Phalen’s tests, and 67% in those that exhibited all three of these clinical features.

In addition to the hands with measured SNC velocities that were included in the above analyses, there were 84 hands in which no signal could be detected when the index finger was tested, indicating extreme impairment of conduction. They included one hand with no numbness/tingling and negative on both Tinel’s and Phalen’s tests, and 26 with extensive median numbness/tingling and positive on both tests. When a random 50% of these 27 hands were added to the group with differences in SNC velocity > 8 m/s, the prevalence of abnormality in hands with no symptoms or signs became 26%, while that in hands with all three clinical features increased to 70%. Furthermore, when abnormality was defined in the same way for the full sample of hands, the prevalence of abnormality in hands with numbness/tingling but negative on both Tinel’s and Phalen’s tests (aggregate categories C, E and H in Table 3) was 25% as compared with 32% in hands with no numbness or tingling (aggregate categories A and B in Table 3).

To check the robustness of our findings, we repeated the analyses for Tables 2 and 3 using as alternative measures of median nerve function: a) distal motor latency; and b) sensory nerve amplitude in the index finger. Results were generally consistent. In particular, there was no increase in geometric mean distal motor latency or reduction in geometric mean sensory nerve amplitude when both Tinel’s and Phalen’s tests were negative.

## Discussion

Our findings suggest that irrespective of symptoms, when both Tinel’s and Phalen’s tests are negative, mean SNC velocity in the median nerve is little different from that in asymptomatic hands. Conversely, abnormal SNC is most likely to be found when both of these tests are positive. In the absence of physical examination, the strongest predictor of abnormal SNC in the median nerve was the occurrence of numbness or tingling in an extensive median distribution with no involvement of non-median parts of the hand. With the methods that we used, a cut-point of 8 m/s in the distribution of the difference between little and index finger SNC velocities appears a reasonable basis on which to define abnormality of median nerve function in epidemiological research.

Our study had the strength of being based on a large sample of patients, and although the response from potential participants was incomplete (73%), and information was partially missing on some others who agreed to take part (for example, because they attended hospital on a day when the research nurse was not present), we have no reason to expect that those included in analyses would have been atypical in the relation of SNC to symptoms and physical signs. Furthermore, the clinical department in which the study was conducted was the only provider of nerve conduction studies for almost all of a local population of some 440,000 people, referrals coming mainly from general practitioners and orthopaedic surgeons. Thus, we would expect that within the age range studied (20–64 years), the associations that we found between clinical findings and SNC will have been fairly representative of those in patients with suspected CTS in the community. However, they cannot necessarily be extrapolated to older populations, in which other causes of neurological symptoms in the hand may be relatively more frequent.

Many of the asymptomatic hands which we studied were in patients with symptomatic CTS in the other hand, and therefore, their prevalence of abnormal nerve conduction may have been higher than that in asymptomatic hands in the general population. To the extent that this occurred, it may have caused us to underestimate the ability of our proposed cut-point of 8 m/s to discriminate between asymptomatic hands and those with typical features of CTS.

Other strengths of our study were the systematic and detailed collection of data on symptoms and the performance of a standardised physical examination. Moreover, by treating SNC velocity as a continuous variable in most of our analysis, we avoided the potential for error from arbitrary dichotomous definitions of abnormality. The distribution of SNC velocities across all of the hands examined was unimodal, with reduced conduction in some hands that were totally asymptomatic. Thus, even if impaired nerve conduction occurred when a clinical test was negative, the finding would not necessarily imply that the test lacked sensitivity.

In a few analyses we did dichotomise SNC velocities, and the predictive values that we then found for abnormality could reasonably be extrapolated to other similar patient populations. However, they would not be expected to apply where the underlying prevalence of abnormality was substantially lower (e.g. when screening a workforce). Nor could sensitivities and specificities necessarily be extended to other settings in which the mix of cases differed (e.g. a higher proportion of hands with borderline as compared with severe abnormality of SNC) or there was a different prevalence of other pathologies that could give rise to the same symptoms. For this reason, and because SNC velocity cannot be regarded as a diagnostic gold standard, we did not calculate sensitivities or specificities.

Report of symptoms, including through use of hand diagrams, may not have been entirely reliable, and it is possible that this contributed to the absence of important associations between nerve conduction and the distribution of pain (Table 2). However, misclassification of symptoms should not have given rise to spurious associations with nerve conduction.

### Implications for clinical practice

In our series of 908 patients, the mean difference between little and index finger SNC velocities in symptomatic hands that were negative for both Tinel’s and Phalen’s tests was no higher than that in hands which had no numbness or tingling (Table 3); and the prevalence of abnormal SNC velocity as we defined it (25%) was rather lower than that in hands without these symptoms (32%). This suggests that unless patients with suspected CTS have other objective evidence of impaired median nerve function (e.g. abnormality on sensory testing or wasting of the thenar muscles), there is little value in referring them for nerve conduction studies if they are negative on both Tinel’s and Phalen’s tests.

Other studies that have examined the utility of Tinel’s and Phalen’s tests in the diagnosis of CTS have generally reported low sensitivity in relation to nerve conduction abnormalities [9,10,12,16-18,21,25-27,31]. However, because SNC velocity can be abnormal in asymptomatic hands, an ideal test would have a sensitivity of less than 100% when assessed against nerve conduction as a standard. In a study which made allowance for this by latent class analysis, the sensitivities of Tinel’s and Phalen’s tests were 0.97 and 0.92 respectively [3].

Among the 788 patients in our case series who underwent physical examination, 228 (29%) were negative for both Tinel’s and Phalen’s tests in both hands. This suggests that screening out patients negative for both of these tests could materially reduce the workload of some neurophysiology services.

### Implications for epidemiological research

A recent review highlighted wide variation in the case definitions that have been used for CTS in epidemiological research [34]. When defining cases for epidemiological purposes, a balance must be drawn between sensitivity and specificity, and the choice that is made will depend in part on the purpose of the research. For example, when estimating population attributable burdens of disease, it may be preferable to adopt a more sensitive and less specific case definition, any inflation of total disease prevalence because of false positives tending to be offset by reductions in estimates of relative risk (because of bias towards the null). On the other hand, for hazard identification and characterisation, a more specific case definition may be advantageous. Another consideration is the types of data that are available. In some studies, information may be obtained only on symptoms or only on symptoms and physical signs.

Our findings suggest that where information is limited to symptoms (e.g. in a survey based on questionnaires), the most specific case definition would be numbness/tingling with an extensive median distribution, and also affecting part-median regions of the hand, but with no involvement of non-median regions. A more sensitive, but somewhat less specific definition would be numbness/tingling with an extensive median distribution, irrespective of other symptoms. We have not found previous studies that classified symptoms exactly as we did, but these conclusions accord broadly with earlier research. Thus, one study found that mean SNC velocity was lower when sensory symptoms occurred in at least three of the four radial digits as compared with when only one or two of these digits was affected [11], and another that neurophysiological abnormality was more prevalent when paraesthesiae were restricted to the median nerve distribution [13]. At the same time, several investigators have observed that patients with abnormal nerve conduction often have sensory symptoms outside the distribution of the median nerve [11,35,36]. Some studies have suggested that report of pain in the hand also has diagnostic value [9,13,30], although an association with impaired nerve conduction has not always been found [14], and there was none in our study.

If Tinel’s and Phalen’s tests can be performed in addition to ascertainment of symptoms, then a relatively specific case definition for epidemiological studies would be numbness/tingling with an extensive median distribution, combined with a positive Tinel’s test and positive Phalen’s test. A more sensitive but less specific case definition would include anyone with symptoms in whom either Tinel’s or Phalen’s test was positive.

In some studies, there may be a need to distinguish between abnormal and normal nerve conduction. While the choice of a cut-point for abnormality will inevitably be somewhat arbitrary, our results suggest that with the method of testing that we employed, a reasonable definition for an abnormal difference between SNC velocities in the little and index fingers sufficient to cause symptoms would be a value of >8 m/s.

## Conclusions

In summary, this relatively large study supports the use of Tinel’s and Phalen’s tests as a filter when referring patients with possible CTS for nerve conduction studies, at least for those of working age. In addition, it highlights the diagnostic relevance of the extent to which numbness and tingling affect the sensory distribution of the median nerve, especially for use in epidemiological research where physical examination and nerve conduction studies are not available. And it demonstrates an approach by which to define a threshold for abnormal nerve conduction that could be used in epidemiological studies. The validity and practical utility of our proposed definition for abnormal median nerve conduction can be tested by exploring whether it distinguishes a group of patients who differ in their risk factors or response to treatment. This is examined in two companion papers [37,38].

## Competing interests

The authors declare that they have no competing interests.

## Authors’ contributions

DC and KTP conceived the study and oversaw its conduct. RVdS and CC contributed to the study design. CL and ECH carried out the data collection and prepared the data for analysis. GN carried out the statistical analysis. DC wrote the first draft of the manuscript. All of the authors contributed to revision and finalisation of the manuscript. All authors read and approved the final manuscript.

## Pre-publication history

The pre-publication history for this paper can be accessed here:

http://www.biomedcentral.com/1471-2474/14/242/prepub

## References

[B1] Claes F Verhagen WIM Meulstee J Current practice in the use of nerve conduction studies in carpal tunnel syndrome by surgeons in the Netherlands J Hand Surg Eur 2007 32E 663 667 10.1016/J.JHSE.2007.09.007 17993428

[B2] Lew HL Date ES Pan SS Wu P Ware PF Kingery WS Sensitivity, specificity, and variability of nerve conduction velocity measurements in carpal tunnel syndrome Arch Phys Med Rehabil 2005 86 12 16 10.1016/j.apmr.2004.03.023 15640982

[B3] LaJoie AS McCabe SJ Thomas B Edgell SE Determining the sensitivity and specificity of common diagnostic tests for carpal tunnel syndrome using latent class analysis Plast Reconstr Surg 2005 116 502 507 10.1097/01.prs.0000172894.21006.e2 16079681

[B4] Witt JC Hentz JG Stevens JC Carpal tunnel syndrome with normal nerve conduction studies Muscle Nerve 2004 29 515 522 10.1002/mus.20019 15052616

[B5] Taylor-Gjevre RM Gjevre JA Nair B Suspected carpal tunnel syndrome. Do nerve conduction study results and symptoms match? Canadian Fam Physician 2010 56 e250 254 PMC2922827 20631260

[B6] Dale AM Descatha A Coomes J Franzblau A Evanoff B Physical examination has a low yield in screening for carpal tunnel syndrome Am J Ind Med 2011 54 1 9 10.1002/ajim.20915 21154516 PMC3624757

[B7] Wilder-Smith EP Seet RCS Lim ECH Diagnosing carpal tunnel syndrome - clinical criteria and ancillary tests Nat Clin Pract Neurol 2006 2 366 374 16932587 10.1038/ncpneuro0216

[B8] Graham B The value added by electrodiagnostic testing in the diagnosis of carpal tunnel syndrome J Bone Joint Surg Am 2008 90 2587 2593 10.2106/JBJS.G.01362 19047703

[B9] Gomes I Becker J Ehlers JA Nora DB Prediction of the neurophysiological diagnosis of carpal tunnel syndrome from the demographic and clinical data Clin Neurophysiol 2006 117 964 971 10.1016/j.clinph.2005.12.020 16516550

[B10] O'Gradaigh D Merry P A diagnostic algorithm for carpal tunnel syndrome based on Bayes's theorem Rheumatology 2000 39 1040 1041 10.1093/rheumatology/39.9.1040 10986313

[B11] Wilder-Smith EP Ng ES Chan YH Therimadasamy AK Sensory distribution indicates severity of median nerve damage in carpal tunnel syndrome Clin Neurophysiol 2008 119 1619 1625 10.1016/j.clinph.2008.03.022 18467170

[B12] Wainner RS Fritz JM Irrgang JJ Delitto A Allison S Boninger ML Development of a clinical prediction rule for the diagnosis of carpal tunnel syndrome Arch Phys Med Rehabil 2005 86 609 618 10.1016/j.apmr.2004.11.008 15827908

[B13] Nora DB Becker J Ehlers JA Gomes I What symptoms are truly caused by median nerve compression in carpal tunnel syndrome? Clin Neurophysiol 2005 116 275 283 10.1016/j.clinph.2004.08.013 15661105

[B14] de Campos CC Manzano GM Leopoldino JF Nóbrega JAM Sañudo A de Araujo PC The relationship between symptoms and electrophysiological detected compression of the median nerve at the wrist Acta Neurol Scand 2004 110 398 402 10.1111/j.1600-0404.2004.00332.x 15527453

[B15] Ferry S Silman AJ Pritchard T Keenan J Croft P The association between different patterns of hand symptoms and objective evidence of median nerve compression Arthritis Rheum 1998 41 720 724 10.1002/1529-0131(199804)41:4<720::AID-ART20>3.0.CO;2-6 9550482

[B16] Gellman H Gelberman RH Tan AM Botte MJ Carpal tunnel syndrome. An evaluation of the provocative diagnostic tests J Bone Joint Surg [Am] 1986 68A 735 737 3722231

[B17] Golding DN Rose DM Selvarajah K Clinical tests for carpal tunnel syndrome: An evaluation Br J Rheumatol 1986 25 388 390 10.1093/rheumatology/25.4.388 3779325

[B18] Kuhlman KA Hennessey WJ Sensitivity and specificity of carpal tunnel syndrome signs Am J Phys Med Rehabil 1997 76 451 457 10.1097/00002060-199711000-00004 9431262

[B19] Bland JDP The value of the history in the diagnosis of carpal tunnel syndrome J Hand Surg Br 2000 25B 445 450 10991809 10.1054/jhsb.2000.0452

[B20] Katz JN Stirrat CR Larson MG Fossel AH Eaton HM Liang MH A self-administered hand symptom diagram for the diagnosis and epidemiologic study of carpal tunnel syndrome J Rheumatol 1990 17 1495 1498 2273490

[B21] Katz JN Larson MG Sabra A Krarup C Stirrat CR Sethi R The carpal tunnel syndrome: Diagnostic utility of the history and physical examination findings Ann Intern Med 1990 112 321 327 10.7326/0003-4819-112-5-321 2306060

[B22] Hems TEJ Miller R Massraf A Green J Assessment of a diagnostic questionnaire and protocol for management of carpal tunnel syndrome J Hand Surg Eur 2009 34E 664 670 10.1177/1753193409105566 19587078

[B23] Bonauto DK Silverstein BA Fan ZJ Smith CK Wilcox DN Evaluation of a symptom diagram for identifying carpal tunnel syndrome Occup Med 2008 58 561 566 10.1093/occmed/kqn123 18796697

[B24] Glass I Ring H Median nerve conduction tests and Phalen's sign in carpal tunnel syndrome Electromyogr Clin Neurophysiol 1995 35 107 112 7781571

[B25] Seror P Tinel's sign in the diagnosis of carpal tunnel syndrome J Hand Surg Br 1987 12-B 364 365 3437206 10.1016/0266-7681_87_90190-2

[B26] Seror P Phalen's test in the diagnosis of carpal tunnel syndrome J Hand Surg Br 1988 13-B 383 385 3249132 10.1016/0266-7681_88_90160-x

[B27] Buch-Jaeger N Foucher G Correlation of clinical signs with nerve conduction tests in the diagnosis of carpal tunnel syndrome J Hand Surg Br 1994 19B 720 724 7706873 10.1016/0266-7681(94)90244-5

[B28] Sawaya RA Sakr C When is the Phalen's test of diagnostic value: An Electophysiologic Analysis? J Clin Neurophysiol 2009 26 132 133 10.1097/WNP.0b013e31819d8046 19279501

[B29] Bridges MJ Robertson DC Chuck AJ Predicting the result of nerve conduction tests in carpal tunnel syndrome using a questionnaire Hand Surgery 2011 16 39 42 10.1142/S0218810411005047 21348029

[B30] Ansari NN Adelmanesh F Naghdi S Mousavi S The relationship between symptoms, clinical tests and nerve conduction study findings in carpal tunnel syndrome Electromyogr Clin Neurophysiol 2009 49 53 57 19280800

[B31] El Miedany Y Ashour S Youssef S Mehanna A Meky FA Clinical diagnosis of carpal tunnel syndrome: Old tests - new concepts Joint Bone Spine 2008 75 451 457 10.1016/j.jbspin.2007.09.014 18455945

[B32] Stata-Corp Stata Statistical Software. Release 11:1 2009 2009 TX: College Station

[B33] Reading I Walker-Bone K Palmer KT Cooper C Coggon D Anatomic distribution of sensory symptoms in the hand and their relation to neck pain, psycho-social variables and occupational activities Am J Epidemiol 2003 157 524 530 10.1093/aje/kwf225 12631542

[B34] Descatha A Dale AM Franzblau A Coomes J Evanoff B Comparison of research case definitions for carpal tunnel syndrome Scand J Work Environ Health 2011 37 298 306 10.5271/sjweh.3148 21301789 PMC3317883

[B35] Clark D Amirfeyz R Leslie I Bannister G Often atypical? The distribution of sensory disturbance in carpal tunnel syndrome Ann R Coll Surg England 2011 93 470 473 10.1308/003588411X586191 21929918 PMC3369333

[B36] Stevens JC Smith BE Weaver AL Bosch EP Deen HG Wilkens JA Symptoms of 100 patients with electromyographically verified carpal tunnel syndrome Muscle Nerve 1999 22 1448 1456 10.1002/(SICI)1097-4598(199910)22:10<1448::AID-MUS17>3.0.CO;2-Y 10487914

[B37] Coggon D Ntani G Harris EC Linaker C Van der Star R Cooper C Palmer KT Differences in risk factors for neurophysiologically confirmed carpal tunnel syndrome and illness with similar symptoms but normal median nerve function: a case–control study BMC Musculoskelet Disord 2013 14 240 10.1186/1471-2474-14-240 23947720 PMC3765327

[B38] Coggon D Ntani G Harris EC Linaker C Van der Star R Cooper C Palmer KT Impact of carpal tunnel surgery according to pre-operative abnormality of sensory conduction in median nerve: a longitudinal study BMC Musculoskelet Disord 2013 14 241 10.1186/1471-2474-14-241 23947746 PMC3765505

